# A study of transfer of information in animal collectives using deep learning tools

**DOI:** 10.1098/rstb.2022.0073

**Published:** 2023-04-10

**Authors:** Francisco Romero-Ferrero, Francisco J. H. Heras, Dean Rance, Gonzalo G. de Polavieja

**Affiliations:** Champalimaud Research, Champalimaud Foundation, 1400-038 Lisbon, Portugal

**Keywords:** collective behaviour, zebrafish, mathematical model, deep learning, software tools

## Abstract

We studied how the interactions among animals in a collective allow for the transfer of information. We performed laboratory experiments to study how zebrafish in a collective follow a subset of trained animals that move towards a light when it turns on because they expect food at that location. We built some deep learning tools to distinguish from video which are the trained and the naïve animals and to detect when each animal reacts to the light turning on. These tools gave us the data to build a model of interactions that we designed to have a balance between transparency and accuracy. The model finds a low-dimensional function that describes how a naïve animal weights neighbours depending on focal and neighbour variables. According to this low-dimensional function, neighbour speed plays an important role in the interactions. Specifically, a naïve animal weights more a neighbour in front than to the sides or behind, and more so the faster the neighbour is moving; and if the neighbour moves fast enough, the differences coming from the neighbour’s relative position largely disappear. From the lens of decision-making, neighbour speed acts as confidence measure about where to go.

This article is part of a discussion meeting issue ‘Collective behaviour through time’.

## 1. Introduction

The study of collective animal behaviour has recently benefitted from a number of technical advances. These include the use of robots (see [1] for a recent review) and virtual reality [2,3]. These two techniques allow the decoupling of interactions to study them causally in a quantitative way. Also, advances in quantitative animal and brain imaging [4–6] are allowing more realistic models of collective behaviour taking into account vision and cognition [7–10].

Deep learning is another technical advance that is affecting all the sciences [11–16]. In the study of collectives, machine learning had been used for a long time [17], but deep learning has managed to give very high accuracies when datasets are large. Applications include tracking in collectives [18,19], posture analysis [20,21] and ethogram production [22–24]. Deep learning applications to collective behaviour are mainly developed and used in laboratory settings, but there is also a migration of techniques to the wild [25].

Deep learning is also allowing one to obtain very accurate models of animals in collectives [26]. However, the usefulness of these models is not obvious as modellers not only want accuracy but also mechanistic insights that can aid the design of new experiments. We propose using a hybrid approach by writing down a mathematical expression for a model and letting deep learning figure out from data those parts we left without a concrete functional decision [26–28]. The parts obtained by deep learning might have many parameters for expressivity, yet a low number of input and output variables. The functions obtained can then be of low dimensionality and therefore easy to plot and analyze. Furthermore, if one requires a simpler symbolic format in terms of traditional functions like ‘sin’ or ‘exp’, deep learning models might still be used as an intermediate step for their good interpolation abilities from data [29].

Here, we extend the toolbox of deep learning applications to help in the study of collective animal behaviour. We built idmatcher.ai as a deep learning tool that uses the results of our deep learning tracking system, idtracker.ai [18], to follow animals across different videos. This ability to follow animals across videos can be used in different types of experiments. For example, it can be used in very long experiments in which it is not possible to record a single video. Instead, videos might be taken at some time intervals and then idmatcher.ai re-identifies the individuals from a video in other videos. It can also be used in experiments in which one wants to identify a subset of the animals with some difference from the rest of the group, e.g. with different ages, experiences or physiological characteristics. Here, we used idmatcher.ai to identify in a video of 15 animals, 5 trained and 10 untrained, which are the ones that correspond to a video of only the 10 untrained ones.

We also developed ReactNet to predict when animals reacted to an external stimulus. The need for ReactNet is less clear as it would seem that a simpler approach of looking at when an animal moves rapidly could solve the problem. We found, however, that animals can react to the external stimulus in several ways, for example increasing velocity, acceleration, angular momentum, or bending the body. It is easier to include all these reaction modes as data to train a network than to come up with all the ways in which a reaction can happen. We used ReactNet to determine in a consistent way when trained animals were reacting to a light being turned on and when the untrained animals started to behave similarly to the trained animals.

To obtain a model for the untrained animals, we used our SocialNet model [26] to explain the behaviour of untrained animals as the focal animal with trained and untrained animals as the neighbours. SocialNet is one such hybrid model in the sense of having a general mathematical structure imposed as our modelling constraints, but which also leaves parts of the expression as low-dimensional functions for deep learning to infer from data. These low-dimensional functions obtained from data as part of the SocialNet model are richer in detail than what one can obtain with simple models. We can then plot two variables of the low-dimensional function, fixing the rest, to check the property of interest. For example, one might want to find out whether interactions in a collective take into account whether a neighbour is in a collision path or whether the repulsion zone depends on how fast an animal is moving [26]. In our case, trained fish reacted to light onset by moving faster. We then queried these low-dimensional functions of SocialNet as to how neighbour speed and acceleration affected the focal behaviour.

We used these three tools (idmatcher.ai, ReactNet and SocialNet), alongside idtracker.ai, to study how information transfer takes place in a group of zebrafish. We mimicked in the laboratory a situation in which some fish in a collective find an interesting location to visit, e.g. a place with food, and the other fish follow them to the food. To have some experimental control, we trained some fish to associate an LED light turning on with food presence. This conditioned them to approach the LED light when it was turned on. We then performed experiments with groups of 15 fish in which 5 of them were trained this way, while the remaining 10 had been desensitized to the light. Using light instead of food allowed us to have more precise control of which animals were reacting, and when. Untrained fish moving to the light must then be influenced by the trained fish and only after at least one of the trained fish reacted to the light. To be able to take advantage of this controlled situation, we used idmatcher.ai to distinguish which of the 15 animals were the 10 untrained ones and which were the 5 trained ones. ReactNet allowed us to be precise and consistent about when each fish was reacting to the light. SocialNet was used to obtain a model for the untrained fish interacting with neighbours. Trained fish reacted to the light with an increase in speed and acceleration. SocialNet shows that untrained fish weigh more any neighbour (mainly frontal ones) that moves quickly and, when moving fast enough, neighbours at any position.

## 2. Methods

### (a) Animal rearing and handling

Fish were raised at the Champalimaud Foundation (CF) Fish Platform according to the housing and husbandry methods integrated in the zebrafish welfare program [30]. Animal handling and experimental procedures were approved by the CF Ethics Committee (CF internal reference 2020/002) and the Portuguese Direcção Geral Veterinária (DGAV reference 0421/000/000/2020) and were performed according to European Directive 2010/63/EU13. We performed all experimental procedures with juvenile zebrafish (28–34 days post-fertilization, dpf) of the Tubingen strain. All experiments were performed in the months of October to December of 2019 at CF (Lisbon, Portugal).

### (b) Experiments

#### (i) Experimental setup

**Figure 1.**
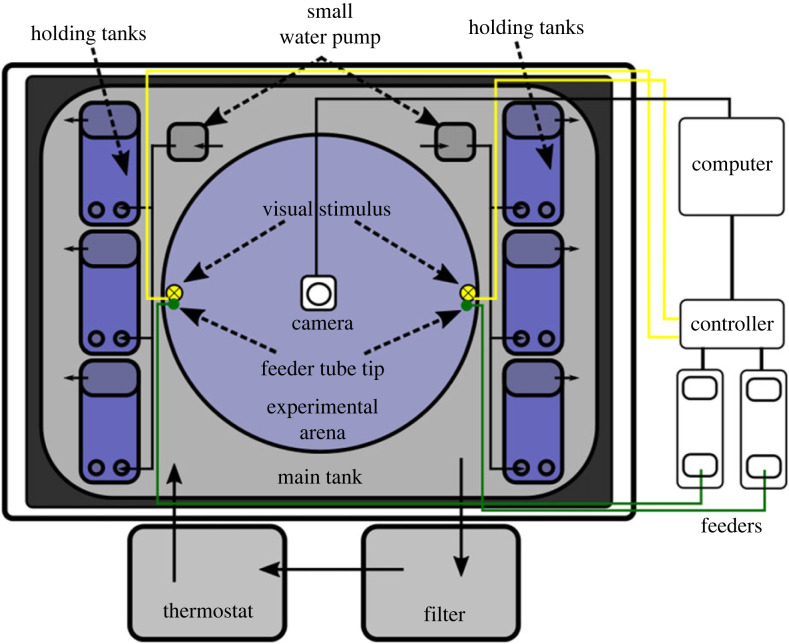
Zenital view of the experimental setup. Text and dashed arrows are the elements of the setup. Solid black lines with arrows indicate water flow direction.

The experimental setup was a modified version of the one described in [18]. Figure 1 shows a diagram of the setup in zenital view. Inside the experimental box, there was a rectangular water tank (main tank) that contained holding tanks and the experimental arena. When not in the arena, the fish were kept in the holding tanks. The main tank had a recirculating water system with ≈300 L of water from the fish facility (pH 7.2–7.4; 1300 μS, 28°C) to avoid changes in water conditions. A thermostat and a filter with a water pump ensured adequate water conditions in the main tank. Smaller water pumps inside of the main tank produced water flow from the main tank to each holding tank. Each holding tank had a baffle that made water flow back to the main tank, keeping the water level in the holding tanks constant.

The illumination of the experimental box was similar to that presented in [18, Supplementary figure 3]. The fish were held in the experimental setup for 7 days in a 14 h/10 h light/dark cycle, using an automatic timer connected to the power source of the RGB and IR strips.

Our experimental procedure required presenting a visible light in localized areas of the arena. For this, we placed two visible white LEDs in opposite sites of the experimental arena at ≈1 cm above the water surface. We placed a second white matte circular tank of the same size as the experimental arena ≈3 cm below to provide an opaque surface where we projected the light coming from the visible light LED. We video-recorded in IR using a IR pass filter (MIDOPT LP715 Near-IR Longpass Filter) in front of the lens of the camera, so only the IR light with wavelengths below ≈715 nm would be captured. When we presented the visible LED in training experiments, we also had an IR LED that activated at the same time and outside of the swimming region of the fish and facing towards the camera. This IR LED, and not the visible LEDs, is the one we can see in the video recordings.

**Figure 2.**
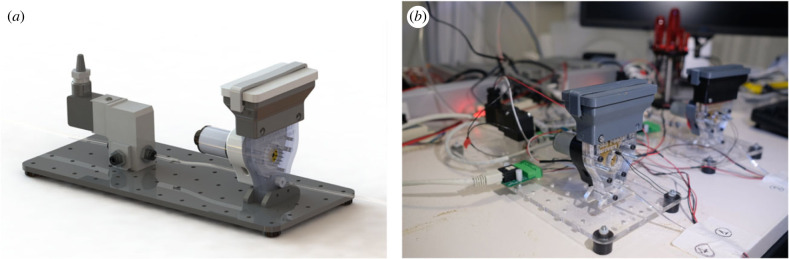
Feeder device. (*a*) Model of the feeder device used to deliver small amounts of food during the training procedure. (*b*) Photo of the two feeder devices outside of the experimental box.

**Figure 3.**
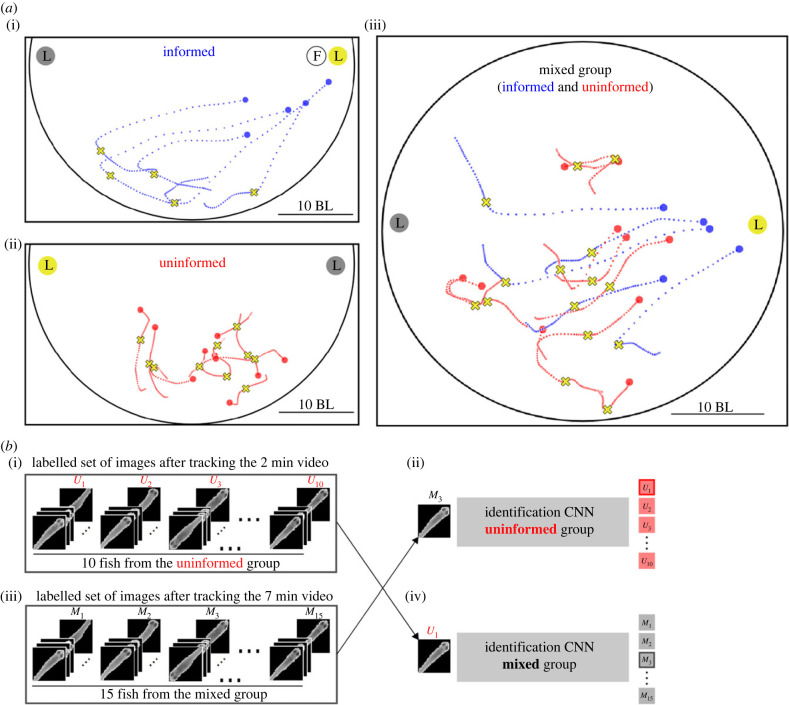
Experiment, tracking, matching and reaction times. (*a*) (i) Trajectories in blue for groups of 5 fish being trained to go to one of the two lights when it is turned on. Training is by associating food (*F*) delivered automatically with a light turning on (L in yellow circle). (ii) Trajectories in red for groups of 10 fish that were desensitized to not react to light. (iii) Experiments consisted of groups of 15 fish mixing 5 informed and 10 uninformed fish. Yellow crosses indicate the times of reaction to the light for each fish, as computed by ReactNet. (*b*) In videos of mixed fish, idmatcher.ai distinguished between those fish that are trained (blue) and those that are untrained (red). It works by re-identifying the fish from a 2 min-long video of only untrained fish in a 7 min-long video of a mix of 5 trained and the 10 untrained fish. First, idtracker.ai is run on each video separately. For video 1, this gives the sets of images for each untrained fish {*U*_1_, *U*_2_, …, *U*_10_}, and the convolutional neural network (CNN; uninformed) to correctly classify these fish. For video 2, it gives the images of the 15 fish {*M*_1_, *M*_2_, …, *M*_15_} and the CNN (mixed) to identify these fish with these labels. Then, idmatcher.ai used CNN (mixed) to re-classify the images {*U*_1_, *U*_2_, …, *U*_10_} into the labels {*M*_1_, *M*_2_, …, *M*_15_} and the CNN (uninformed) to re-classify images {*M*_1_, *M*_2_, …, *M*_15_} into labels {*U*_1_, *U*_2_, …, *U*_10_}, and when both procedures coincided, we had a match. BL, body lengths.

To facilitate the delivery of the food in the water during the training of the informed fish (see §2(b)(ii)), we included in the setup two feeders designed and custom-built by the CF Hardware platform (figure 2; contact at https://www.cf-hw.org/ for further information). Each feeder consisted of a chamber where the food (GEMMA Micro 300) was stored, a motor with a cogwheel, a valve and a set of tubes. The feeders were placed outside experimental box, and a set of tubes directed the food from the feeder to the area in the experimental arena next to where the visible LED was located (figure 3). When a feeder was activated, the motor rotated, depositing a small amount of food into the tube. Then, the valve released a puff of compressed air that moved the food until the end of the tube and deposited the food on the surface of the water in the experimental arena. We ensured that the food was deposited in the area where the light coming from the LED was projected.

We controlled the visible LED, the IR LED and the feeder using the HARP Behaviour controller (https://www.cf-hw.org/harp/behavior) through the Bonsai software [31].

#### (ii) Training and desensitization

Here, we describe the procedures that we followed to train groups of juvenile zebrafish (28–35 dpf) to move rapidly to a visible light in the arena when it turned on. These trained fish are called the informed group. We also desensitize other fish so that they do not respond the light, and will call these fish the uninformed group.

The training and desensitization procedures lasted 7 days. The procedures began with 3 days of food deprivation followed by 4 days of training. All procedures started on a Friday, with the fish being food deprived during the weekend, then the training and desensitization sessions started on Monday. For simplicity, we call a training and desensitization session just a *training session*. The group condition (i.e. informed or uninformed) determines if it was a training or a desensitization session, respectively.

On the first day (Friday), at ≈2 pm, we prepared 4–6 groups of 10 juvenile zebrafish (28–29 dpf), each in standard 3.5 L holding tanks. We introduced these holding tanks into the main tank of the experimental setup (see §2(b)(i)) and we deprived the fish of food for ≈60 hours.

On the fourth day (age 31–32 dpf), we started the training and desensitization. We performed three training sessions per day in the following time intervals: 9 am–11 am, 1 pm–3 pm and 5 pm–7 pm. We chose these intervals to match the same feeding schedule of fish of the same age held at the CF Fish Platform. Each group underwent a total of 10 training sessions. This corresponds to 3 full days (Monday, Tuesday and Wednesday), with three sessions each day and a last training session on the seventh day (Thursday) of the procedure. In each of the intervals, we trained four to six groups. We randomized the training order of the groups in each of these intervals.

The purpose of this training procedure was to create mixed groups of 15 fish each made up of 5 informed fish and 10 uninformed fish. For this reason, before the last training session, we separated each group of 10 informed fish into two subgroups of 5 informed fish. Thus, in the last training session, each group of informed fish had 5 animals.

At the end of each training session, we returned the fish back into their corresponding holding tank. The fish stayed in the holding tank until the following training session or until we performed the experiment using a mix of informed and uninformed fish (see §2(b)(iii)). To remove any possible bias due to the position of the animals in the main tank, we randomized the position of the holding tanks in the main tank after each training session. In total, we desensitized 22 groups of 10 uninformed fish and trained 22 groups of 5 informed fish.

Next, we give details of the training and desensitization procedures for the uninformed and informed groups, respectively. Before each training session, we rinsed the experimental arena with water from the same holding system where the fish are housed in the CF Fish Platform. Then, we filled the tank with ≈5 L of the same water, which corresponded to ≈2.5 cm of depth in the centre of the experimental arena (the body height of a juvenile fish at this age was ≈0.3 cm). We then introduced the fish of the corresponding group in the experimental arena with a standard fish net.

For the uninformed groups, we manually spread food (≈32 ± 3 mg of GEMMA Micro 300; mean ± SD) as uniformly as possible across the arena using a fish-food plastic spoon. After spreading the food, the fish consumed it. After ≈1 min, fish stopped displaying feeding movements and started swimming together. At this point, we started the desensitization session. During 8 min, we sequentially presented the visible lights (see §2(b)(i)), one at a time. We call the presentation of one of the visible lights a *trial*. The sequence of trials followed a pseudo-randomized order while ensuring that the same light was not activated for more than 2 trials in a row. Each session contained 14 ± 3 (mean ± SD) trials, and in each trial, the light was presented for 5 ± 3 s. Consecutive trials were spaced by 23 ± 16 s.

For the informed groups, we did not spread any food at the beginning of the training session. Before the training session, we allowed animals to habituate for ≈1 min. Then, we started the training session. During 8 min, we sequentially presented the visible lights one at a time in a sequence of trials. The light that was turned on was chosen pseudo-randomly. Each session consisted of 9 ± 3 trials. In each trial, if during the first ≈5 s after the light onset some of the animals moved closer to the on light, we rewarded such movement by delivering food (≈5 ± 2 mg of GEMMA Micro300) in that region of the arena using the custom feeder devices (see figure 2) and turned the light off after ≈10 s. Otherwise, we turned the light off. On average, in each trial, the light was on for 12 ± 6 s. Consecutive trials were spaced by 33 ± 17 s. To shape the behaviour of the animals, during the first two training sessions, the lights were turned on when the fish were already swimming towards them, and food was deposited even if the light was off.

#### (iii) Experiments in groups mixing informed and uninformed fish

We used groups of 15 fish, 5 informed and 10 uninformed fish. Henceforth, we will refer to this experiment as ‘the experiment’ and the previous training/desentization procedures as ‘training’. During the experiment, we never delivered any food.

We performed the experiment on the same day as the last training session (Thursday), between 12 pm and 3 pm. At this point, the fish were 34–35 dpf. During this time, we performed four to six experiments of 10 min each.

We first introduced into the arena the group of 10 uninformed fish and allowed them to swim freely for ≈2 min. During these ≈2 min, we did not deliver food anywhere nor did we turn on either of the lights. After these ≈2 min, we introduced into the arena the group of 5 informed fish and allowed them to swim freely together with the 10 uninformed fish for ≈1 min.

During the next 7 min, we ran repeated trials of turning a light on for 5 ± 2 s, with 44 ± 17 s between trials. Which light we turned on was chosen pseudo-randomly, similarly to the training. In total, we performed 22 experiments of mixed groups.

### (c) Animal tracking

We video-recorded all the training sessions and all the experiments. We extracted the trajectories of the animals in all the videos using idtracker.ai [18]. When tracking the 10 min videos of the experiment, we used the tracking interval feature of idtracker.ai and tracked the first 2 min (where only the 10 uninformed fish were present) and the last 7 min (once the 5 informed fish had been added to the group) independently but with the same animal detection parameters for image segmentation of animals from the background.

If the estimated accuracy reported by idtracker.ai at the end of the tracking was below 99.5%, we re-adjusted the animal detection parameters and tracked the video again. The average estimated accuracy of the tracking for the videos corresponding to the training procedures was 99.9±0.2%, for the first 2 min of the experiment was 99.98±0.01% and for the last 7 min 99.99±0.01%.

### (d) idmatcher.ai: matching individuals in different videos

For each mixed group of fish with 10 uninformed fish and 5 informed fish, we had a 2 min-long video of only the 10 uninformed fish (video 1) and a 7 min-long video of the 15 animals (video 2). Our task was to identify which 10 fish in video 2 were the 10 uninformed fish in video 1. We developed idmatcher.ai as a Python package with tools to match the identities of the animals in two videos tracked with idtracker.ai.

Given two videos, videos 1 and 2, idtracker.ai fits a convolutional neural network (CNN) that identifies the individuals of video 1 (idCNN_1_) and obtains a set of images for each individual of video 1, and similarly for video 2; see figure 3. idmatcher.ai relabels the images of video 1(2, respectively) using the labels of the idCNN of video 2(1). This results in the two matrices **M**^12^ and **M**^21^, respectively. The matrix element $M_{ij}^{12}$ is the number of images labelled with identity *j* using the idCNN from video 2 that are labelled with the identity *i* by the idCNN of video 1, and analogously for $M_{ij}^{21}$. For each matrix **M**^*ab*^, we compute the cost matrix **C**^*ab*^, its elements defined by $C_{ij}^{ab}=1-M_{ij}^{ab}/\sum_{\;j}M_{ij}^{ab}$, where *ab* = 12 or 21. To find the optimal match of identities, we solve the generalized linear assignment problem (with different numbers of rows and columns) with the two cost matrices **C**^12^ and **C**^21^ independently. We used the implementation of the Jonker–Volgenant algorithm [32] from the Scipy Python library.^1^ If the assignment was equivalent in both directions, we considered that the matching was successful. idmatcher.ai found a successful match for 20 out of 22 experiments. We only used these 20 videos for analysis and modelling. We performed the following validation of idmatcher.ai. Each of these 20 videos was recorded continuously. The first 2 min had only the 10 uninformed fish, and then we added the 5 informed fish into the arena. The way we introduced these new five animals did not disturb the images to preclude following the fish visually and check that the matching done by idmatcher.ai was correct.

### (e) Light onset detection

As explained in §2(b)(i), the experimental arena included an IR LED that was activated at the same time as the visible LED. We used the signal from this IR LED to obtain the precise time for when the LED light turned on. We manually annotated the location of each IR LED in each video, and the system automatically created a region of interest (ROI) of 50 pixels around the location of the IR LED. We computed the average intensity of the pixels in this ROI for all frames in the video. This time series of the average intensities showed clear changes when the IR LED was active. To remove background noise, we convolved the time series with a kernel of three frames. Then we computed the derivative of such signal by taking finite differences. The distribution of the derivative had three modes. Very high positive values indicated the onsets of the IR LED, very low negative values indicated the moment when the IR LED was turned off. Values around 0 were the changes due to background noise. We used two thresholds to automatically detect the high transients. The thresholds were determined by creating a histogram of the finite differences with 10 bins. The lowest side of any bin with zero counts was used as the lower threshold, while the highest edge of any bin with zero counts was used as the higher threshold. We labelled the onset of the IR LED as the frames where the derivative of the average intensity time series was higher than the maximum threshold. Note that we properly corrected the length of the time series after the convolution and the computation of the derivative, so that the detected frames matched the first frame where the IR LED was active in the video for each trial.

### (f) ReactNet: detection of fish reactions to light

We trained a neural network, which we named ReactNet, to determine when, and if, each animal reacts to the light turning on.

#### (i) Input and output

The input to ReactNet is a multivariate time series with the following components: position in the plane, velocity in the plane and acceleration in the plane, and finally, a binary flag reflecting whether the animal is informed or uninformed, as informed animals likely react differently to the stimulus. To facilitate learning, the trajectories of each trial are rotated and displaced so that the active feeder is located at the origin, and the centre of the arena is on the positive *x* semi-axis. The output of the network is an approximation to the probability that the light is on.

The trajectories were chosen such that they were contained within trials; the light never went from on to off during the time of a single trajectory. Some data augmentation was performed during training by rotating the trajectories by a small random angle around the origin.

#### (ii) Architecture

The structure of ReactNet is a five-layered gated recurrent unit ([33], a type of a recurrent neural network (RNN)) with 100 hidden dimensions, followed by a cumulative max layer and a logistic activation function. This structure forces the network to have two useful properties: (i) each component of the output depends only on past points of the trajectory and (ii) output of the network monotonically increases in time.

#### (iii) Training ReactNet

To train ReactNet, we used a total of 454 trials. The trials were of two classes. The first class was data from sessions where all fish were informed and had already learned to react to the stimulus (i.e. taken from the last two training sessions). The second class was data where informed and uninformed fish were mixed together in the arena (the experiment). Among these trials in this second class, we selected four trials for validation and four trails for testing. This left 446 trials to build the training dataset.

During training, we forced the output to approximate a target one-dimensional time series of the same length. The target time series contains zeros until two frames after light turned on, and ones thereafter. We chose this margin (two frames, 62.5 ms) because we did not expect any meaningful reaction to happen earlier. We trained the network output to approximate the target by defining a loss and using a modified stochastic gradient descent (Adam [34], learning rate 5 × 10^−5^) on minibatches of size 50 to modify the parameters, as is standard in deep learning. The loss was a weighted sum of the cross-entropy losses in each frame between the output of the network and the target. The weighting was used to counterbalance the bias of the training data (e.g. in most examples, the light had not turned on yet in the first frame but had already turned on in the last frame).

We monitored the loss on the validation dataset to detect overfitting. We performed early-stopping if validation loss had not decreased for 500 consecutive epochs. In any case, we stopped training after 2500 total epochs. Finally, we selected the model that minimized validation loss.

#### (iv) Building a classifier from the trained model

After training, the output of ReactNet approximates the probability of the light being on at each frame conditioned on the behaviour of a given fish. To calculate each prediction, the network can only use information from the past of the same trajectory. Therefore, if ReactNet outputs that it is likely that the light is on at a given frame, it is because the trajectory up to that point showed evidence of the light being on; i.e. evidence of some reaction (direct or indirect) to the light by the animal.

ReactNet outputs a continuously valued real number between 0 and 1. We built classifiers from the output of ReactNet by choosing a fixed threshold, such that outputs above this threshold corresponded to prediction of the light being on and outputs below this threshold predicted that the light was still off. We presented videos to a human with the predicted reaction times at different values of the threshold and asked them to report estimated false positives and negatives for each threshold value. We chose the threshold value of 0.7 as it gave the lowest sum of false positives and negatives.

Any threshold that we could choose allows ReactNet to classify the frames into those where the prior trajectory had shown that the animal had already reacted (‘having reacted’) and frames where the prior trajectory had not yet shown the animal to have reacted (‘not reacted’). As the output is monotonically increasing in time, any frame classified as ‘having reacted’ is followed only by frames with the same classification. We consider the first of such frames as the frame where the animal reaction happened. If no such frame exists, or if it occurs later than 64 frames (2 s) after the stimulus onset, we concluded that the animal had not reacted during the trial.

#### (v) Using the classifier on all data

To obtain the reaction times used in the subsequent analysis, we applied the trained ReactNet to the whole dataset. To obtain data for this application, we cut all trajectories using a fixed window starting 15 frames before the stimulus onset and with a total length of 192 frames.

### (g) SocialNet: modelling animal interactions

To capture the rules of interaction, we used a model that we developed using experiments of large groups of fish moving freely, which we call SocialNet [26]. Here, we used the same modelling assumptions and architecture, but we retrained SocialNet on the present data for the animals that were desensitized only.

#### (i) Input and output

SocialNet is designed to solve the following binary classification problem: given the instantaneous dynamical properties of a focal fish and its *N* closest neighbours, will the focal fish turn right or left in the next 1 s? Dynamical properties come in two classes, asocial and social. Asocial properties refer only to the focal fish and include velocity and acceleration perpendicular to the velocity,

2.1 $$\alpha =\{v,a_{⊥},\ldots \}.$$

Social information consists of the location, velocity and acceleration of neighbour *i*

2.2 $$\sigma_{i}=\{x_{i},y_{i},v_{i},\theta_{i},a_{x,i},a_{y,i},\ldots \},$$

in an instantaneous stationary frame of reference, centred on the focal fish and with the positive *y* semi-axis co-lineal with the focal fish velocity.

The network outputs the logit, *z* = *z*(*α*, *σ*_1_, …, *σ*_*N*_), from which we calculate the turning probabilities using a logistic function *p* = 1/(1 + e^−*z*^).

#### (ii) Architecture

The model assumes that the focal animal interacts with each neighbouring animal *i* via a pair-interaction function $\Pi_{A}(\alpha ,\sigma_{i})$. This assumes the same functional form for all pairwise interactions, but the output value of the interaction differs because the values of the neighbour’s dynamic variables *σ*_*i*_ can differ across neighbours.

The focal interacts with its *N* closest neighbours, weighting differently each neighbour depending on the neighbour’s behaviour. To determine the weight of each of these interactions, SocialNet has a positive weight function *W*(*α*, *σ*_*i*_). The final weight is normalized by $\sum_{j}W(\alpha ,\sigma_{j}),$ so the weight for each neighbour is a value between 0 and 1. Finally, the output logit is determined as follows:

2.3 $$z=\sum_{i=1}^{N}\Pi_{A}(\alpha ,\sigma_{i})\frac{W(\alpha ,\sigma_{i})}{\sum_{j}W(\alpha ,\sigma_{j})}.$$

The functions $\Pi_{A}$ and *W* are each implemented as a neural network. $\Pi_{A}$ has three fully connected hidden layers of 128 neurons each, with rectified linear unit (ReLU) activations. Its final layer has only one neuron yielding the logit *z* of the focal moving to the right in the next 1 s. $\Pi_{A}$ is anti-symmetrized with respect to neighbour relative position *x*.

*W* has three fully connected hidden layers of 128 neurons each and ReLU activations. The final single-neuron layer has an exponential activation function. The output of *W* is symmetrized with respect to a change in sign of the neighbour’s *x* positional coordinate (i.e. symmetrized with respect to reflection around the *y* axis, or the direction of the velocity of the focal). The output value is forced to be positive by using an exponential activation function.

#### (iii) Training SocialNet

We trained SocialNet to minimize the binary cross-entropy loss on a training set using stochastic gradient descent and performed early-stopping and model selection using a separate validation dataset. We then report performance on a held-out test dataset.

We trained SocialNet for *N* = 9 neighbours, a number that is enough both for videos of 10 uninformed fish and of 15 mixed fish. After training, we apply the model to a mixed group of 15 fish using the same modules but this time with *N* = 14.

In total, we used trajectories of 20 videos of 2 min (of uninformed fish, before adding informed fish) and 159 trials of 10 s around the light onset (of mixed groups). The training set contained the trajectories from 100 trials and all trajectories from the twenty 2 min videos. The validation set contained 10 different trials, and the remaining 49 trials formed our test set. We also tested that only using light-off data in training did not change the results (not shown).

## 3. Results

### (a) A setup to study interactions using idtracker.ai and idmatcher.ai

In our previous model of interactions among zebrafish in large collectives, which we called SocialNet, we had already found that the most important variables to predict the behaviour of each fish are its velocity and its acceleration perpendicular to its own velocity [26]. The influence of neighbours came in second place, and it was their position relative to the focal that was the most important of the neighbour’s variables, followed by the velocity of the neighbour, and to a lesser extent their acceleration. While neighbour velocity and acceleration had a small contribution to overall accuracy, we reasoned that at key decision times their impact can be important in the group dynamics. Specifically, we hypothesized that animals with some information about what to avoid or where to go could be moving faster and, if velocity played a role in interactions, any animal in the group would be more influenced by them than by the other animals. This hypothesis was consistent with the reasoning in our previous Bayesian models [35,36], according to which each animal uses the behaviours of others to better estimate events in the world, but those models were tested in very simple decision setups in which velocity could not play a role. Elegant alternative explanations exist, e.g. fish could still be influenced by the mean of neighbours’ locations and when a subset of the agents go to a specific place they would then bias the group to move towards that place.

**Figure 4.**
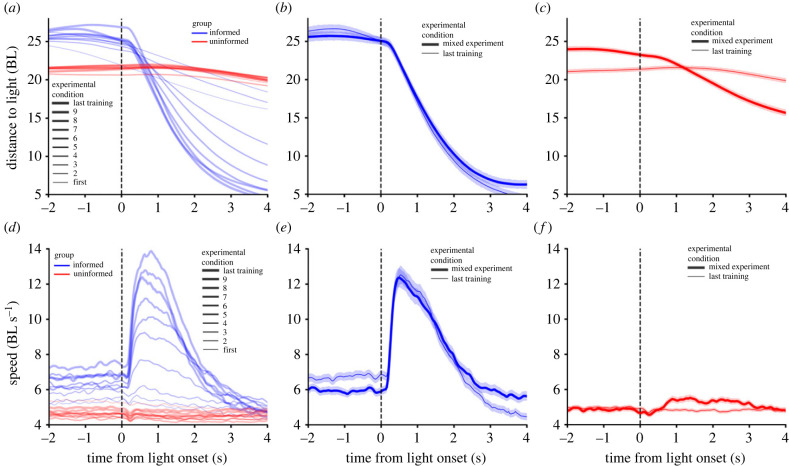
Behaviour during training of informed and uninformed fish, and behaviour when mixed. (*a*) Distance from the fish to the light for nine consecutive training sessions (blue for informed fish and red for uninformed). (*b*) Distance from the informed fish to the light in a mixed experiment (thick line) and in the last training session before the mixed experiment (thin line). (*c*) Same as (*b*) but for the uninformed fish. (*d*–*f*) are as (*a*–*c*) but for speed instead of distance. BL, body lengths.

We used the circular set-up in figure 3*a* ((i,ii) half of the set-up and (iii) full circular setup). At two opposite sides of the setup, we had lights and food dispensers, and we could turn the light on and deliver food from a distance automatically (see §2(b)(i)). A group of five fish were trained to move to the light by associating food presence with light, for both lights (figure 3*a*, (i) blue lines; see §2(b)(ii) for details). With every training session, these fish would move closer to the light (figure 4*a*) and faster (figure 4*d*). These effects and those in the rest of the text have *p* < 0, 001 unless reported otherwise. The fish started to move at a higher velocity at ≈250 ms after light onset and obtained its peak by ≈500 ms, and after 2 s, the velocity was lower than baseline (not shown) as fish reached the light (figure 4*d*).

We call informed fish the fish that we trained to move to the light. We also wanted another 10 fish not to move to the light (uninformed fish), and for this, we trained them not to associate food presence with the light being on (figure 3*a*(ii), red lines; see §2(b)(ii) for details). The uninformed fish showed no approach to the light when it went on (figure 4*a*,*d*, red lines).

We then mixed together the 5 informed and the 10 uninformed fish, and a pseudo-random number determined which of the two lights was turned on, but no food was delivered (figure 3*a*(iii)). We expected that the informed fish would move towards the light, and we could then study the interaction variables that made the uninformed fish follow them to the light. We used 20 different mixed groups of 15 fish.

We built idmatcher.ai to distinguish the informed and uninformed fish in the mixed group experiments. This tool allows matching the identities of animals between two different videos. In our case, the two videos were gathered in the following way. We first introduced the uninformed fish in the tank and recorded them swimming freely for 2 min (let us call this video 1). Then, we introduced the informed fish into the tank, allowed them to swim freely with the uninformed fish for 1 min and then recorded a second video of the experiment for 7 min (video 2).

The task of idmatcher.ai consists of identifying which animals of the mixed group (video 2) are uninformed fish (video 1). idmatcher.ai uses the result of tracking each animal in the two videos with our tool idtracker.ai ([18]; see also §2(c)). idtracker.ai gives us for video 1 a set of images for each uninformed fish {*U*_1_, *U*_2_, …, *U*_10_} (figure 3*b*(i)) and a CNN that correctly classifies each of the images of the uninformed fish (figure 3*b*(ii)). Similarly, for video 2, idtracker.ai gives a set of images for each of the animals in the mixed group {*M*_1_, *M*_2_, …, *M*_15_} (figure 3*b*(iii)) and a CNN that correctly classifies each image of these fish as a different member of the mixed group (figure 3*b*(iv)).

idmatcher.ai then takes the images from the 10 uninformed animals in the first video, with labels {*U*_1_, *U*_2_, …, *U*_10_}, and asks how the CNN obtained for the mixed group re-identifies them. For example, in figure 3*b*, the images of uninformed fish *U*1 are re-identified as fish *M*3. idmatcher.ai also takes the sets of images from the 15 mixed animals in video 2 and asks how the CNN obtained for the uninformed fish would re-classify them. For example, in figure 3*b*, the images from fish M3 are re-identified as fish U1. When there is an agreement between the re-assignments in both directions, as it is the case in the figure, we say we have a match (see §2(d) for details).

Once we had the informed and uninformed fish identified, we could check whether the mixed experiment worked as intended. Informed fish approached the light when it turned on (figure 4*b*,*e*, thick line) in a way that was very similar to how they approached it in the last training session (figure 4*b*,*e*, thin line). On the other hand, the way the uninformed fish behaved was different from their training sessions. Now they approach the light (figure 4*c*, thick line), and the mean velocity values after the light is on are also higher (figure 4*f*, thick line). However, the mean velocity values reach a peak in time at very small values (5.5 BL s^−1^) compared to the mean velocity values of the informed animals (12 BL s^−1^). One possible explanation for why these mean speeds differ is that the individual speeds of the uninformed fish are simply much lower than those of the informed fish. If this explanation is the correct one, the experiment would not have worked as intended, as there would be a much smaller reaction of uninformed fish than expected.

A second possible explanation is that the real speeds of the uninformed and informed fish are similar, but the reaction times of uninformed fish are more variable, resulting in a low value of the mean speed in time. To decide which of the two explanations is correct, we performed an analysis taking into account reaction times.

### (b) Reaction times to light using ReactNet

**Figure 5.**
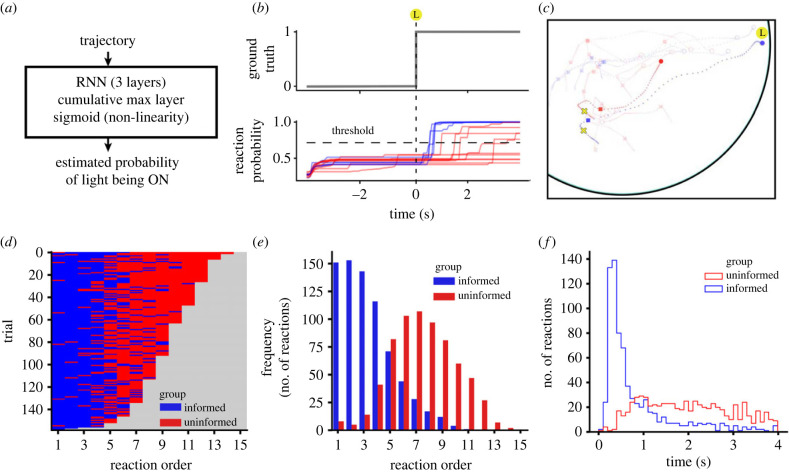
Reaction times computed by React. (*a*) ReactNet is a RNN that takes as input the trajectory of a single fish and outputs the probability that the light is on. (*b*) Example of ReactNet output for a mixed group. Blue lines are probability of light on for the informed animals and red lines for uninformed animals. (*c*) Trajectories are marked with a yellow cross at the reaction time. (*d*) Reaction order for the 160 trials of mixed groups. Trials are depicted from low to high number of responses. (*e*) Number of reactions (of the total 1650 reactions) in which a given reaction order corresponds to an informed or to an uninformed fish. (*f*) Number of reactions in time.

Fish can react to the light with an increase in speed, acceleration or angular momentum or, more generally, a combination of the three. One possibility for detecting reactions would then be to use a threshold value on one or several of these variables [37–39]. As the precise combination of variables can be difficult to obtain, we followed a more agnostic approach of training a recurrent neural network (RNN) that we call ReactNet (figure 5*a*; see §2(b) for details). ReactNet finds the time of reaction of a fish by finding when the trajectory of the fish allows you to correctly predict that the light is on.

ReactNet is a RNN that takes as input the trajectory of an animal and outputs the probability that the light is on (figure 5*a*). After the light is on, this reaction probability increases to values close to 1 before 1 s for the informed individuals (figure 5*b*, blue lines). The uninformed individuals have more variability and reach high values within 1–4 s after the light is on (figure 5*b*, red lines). A threshold value for the probability of 0.7 was chosen to define a reaction time with a balance of false positives and negatives. Yellow crosses in figures 3*a* and 5*c* indicate the point of the trajectory at which a fish reacts to the light, according to ReactNet.

Figure 5*d* gives the reaction order of the informed (blue) and uninformed (red) fish for the 160 trials conducted with 20 different groups of fish. The trials are here depicted from those with the least fish reacting (2) to those with the most fish reacting (14). In all trials, the informed fish (figure 5*d*, blue) tended to react before the uninformed fish (figure 5*d*, red). This raw data of the different trials can be represented more compactly as the number of reactions of informed or uninformed fish at different positions in the order of reactions (figure 5*e*). When presented in real time and not as reaction orders (figure 5*f*), one can see that most reactions of informed fish happen before 1 s and of uninformed fish after 1 s. The first informed fish reacts at 0.5 ± 0.16 s (mean ± std) and the first uninformed fish at 1.2 ± 0.7 s.

We are now in a position to revisit the problem of why, in mixed experiments, the mean velocity of the uninformed fish (figure 4*f*, thick line) was so much smaller than that of the informed fish (figure 4*e*, thick line). For this, we plot the mean velocity of informed and uninformed fish not in real time but using as time origin the time of reaction to the light. In this way, we found that the velocities of both informed and uninformed fish are high, above 10 body-lengths per second (BL s^−1^). As the reaction times of uninformed fish are very variable (figure 5*f*, red line), the resulting mean velocity in real time for informed fish went down to the very small values of 5.6 BL s^−1^ in (figure 4*f*, thick line). Still, note that the informed fish can reach speeds of up to ≈18 BL s^−1^ and uninformed fish up to ≈12 BL s^−1^.

### (c) Variables of interaction using SocialNet

**Figure 6.**
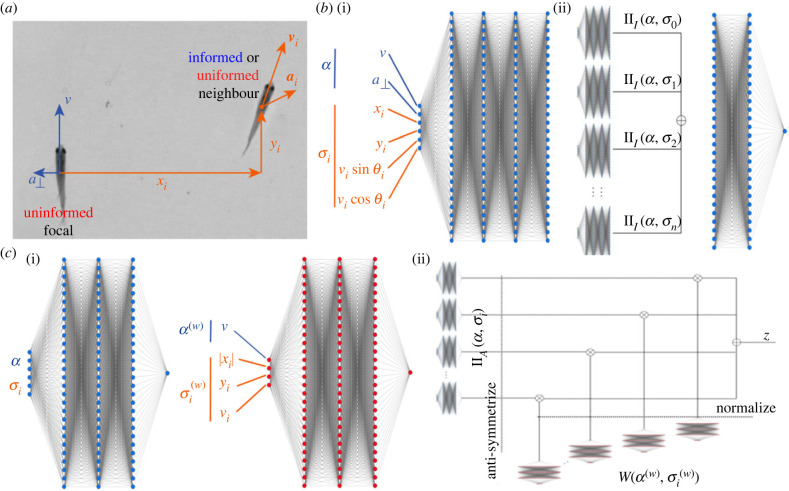
Architectures for the interaction network and SocialNet. (*a*) Variables attempted as input for uninformed focal and for neighbour. (*b*) Pair-interaction module (i), with the variables of the uninformed focal, *α*, and the variables of the *i*th neighbour, *σ*_*i*_ as inputs. Complete interaction network (ii) with 14 pair-interaction modules. Their 128 outputs are added element-wise and processed in a two-layer network to give the output *z*. (*c*) The two modules of SocialNet. The pair-interaction module (i) models the interaction of focal and neighbour *i*, and the aggregation module (ii) gives a positive weight that measures the importance of neighbour *i* in the final output *z*. The complete SocialNet makes a product of the pair-interaction module for neighbour *i* with the aggregation module for each neighbour *i* and sums the results of this for the 14 neighbours to give the output *z*.

We wanted to find the interaction variables among fish, i.e. we wanted to find which variables of the uninformed focal and its neighbours would predict the probability that the uninformed focal is going to turn left or right in the next second. The same approach can be used including other output variables, but we chose moving right for simplicity in posterior analysis. As input variables, we considered both focal and neighbour variables (figure 6*a*). For the focal variables, we considered the focal speed and the focal acceleration,

3.1 $$\alpha =\{v,a_{⊥}\}$$

and, for the variables of neighbour *i*, its location, velocity and acceleration,

3.2 $$\sigma_{i}=\{x_{i},y_{i},v_{i},\theta_{i},a_{x,i},a_{y,i}\},$$

in an instantaneous frame of reference that is not moving, centred on the focal fish and with the positive *y* semi-axis co-lineal with the focal fish velocity.

First, we used the data from mixed experiments to obtain a reference model, which we call the interaction network. This model was used for its flexibility, and the idea was to find some reference accuracy even if it is from an opaque model, figure 6*b*. The interaction network assumes that the focal animal interacts with other animals with a pair-interaction function, $\Pi_{I}(\alpha ,\sigma_{i})$ (figure 6*b*(i)). The complete model has one of these functions for each of the 14 neighbours, each with 128 outputs, then sums all 14 of these outputs element-wise and finally processes the sum to give the logit output (figure 6*b*(ii)). From the logit output *z*, we calculate the probability of turning right using a logistic function *p* = 1/(1 + e^−*z*^).

**Figure 7.**
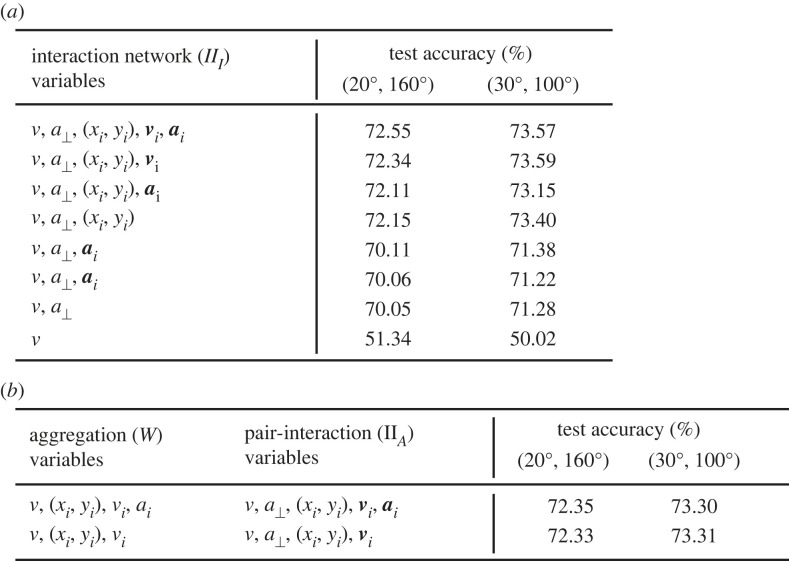
Test accuracies for the interaction network and for SocialNet. (*a*) Variables attempted as input to the interaction network, and test accuracies for cases in which the fish turns an angle of 20−160° or an angle of 30−100°. (*b*) As in (*a*) but for SocialNet.

We obtained the accuracy of the interaction network for test data and for different input variables (figure 7*a*). The focal variables are more impactful on the accuracy and, from the neighbours, their position is the most important variable followed by the speed. Acceleration seems to be less important, and depending on the focal turning angles considered, adding acceleration could worsen the model’s accuracy. We also tried even more flexible models, e.g. all-to-all networks, but their accuracies were lower as the number of parameters is large for the amount of data.

The interaction network, however, is not a very interesting model to obtain further insight on the fish interactions. If we consider 6 input variables per neighbour and 14 neighbours, the complete model is a function of 6 × 14 = 84 variables, too large for analysis. Its relevance is that it gives a reference accuracy that should be our target when considering less flexible models.

We considered SocialNet, a model made up of only two types of modules of low dimensionality (figure 6*c*(i); see our freely moving results in [26] and §2g). The first module is a pair-interaction function similar to the one we considered for the interaction network but this time outputting a single value (figure 6*c*(i)left). This function $\Pi_{A}$ describes how the uninformed focal interacts with one neighbour. There are 14 of these pair-interaction modules, one for each neighbour. Although these 14 modules are identical, each gives a different output because the values of input variables are different for each of them. These 14 values are then weighted into a final decision using a second module (figure 6*c*(i), right), which we call the aggregation weight *W*. This second module takes as input different sets of focal and neighbour variables, *α*^*w*^ and $\sigma_{i}^{w}$ respectively.

The logit output *z* can then be written in terms of the outputs of the two modules, $\Pi_{A}(\alpha ,\sigma_{i})$ and $W(\alpha^{w},\sigma_{i}^{w})$, as follows:

3.3 $$z=\sum_{i=1}^{14}\Pi_{A}(\alpha ,\sigma_{i})\frac{W(\alpha^{w},\sigma_{i}^{w})}{\sum_{j}W(\alpha^{w},\sigma_{\;j}^{w})},$$

also depicted using networks in (figure 6*c*(ii).

For the second module to act as an aggregation weight, *W* needs to be positive. Note that the input variables for *W* need not be the same ones as for the interaction function $\Pi_{A}$ and, for that reason, we use a superscript, *α*^*w*^ and $\sigma_{i}^{w}$. A second condition for *W* to be used as an aggregation weight is that it is normalized by $\sum_{j}W(\alpha^{w},\sigma_{\;j}^{w})$, so each neighbour is weighted by a value between 0 and 1.

For a better use of the data to obtain the SocialNet model, we assumed that fish are left–right symmetric. For the weight function, this is done simply by using as input the absolute value of the position *x*_*i*_ of neighbour *i*. For the interaction function $\Pi_{A}(\alpha ,\sigma_{i}),$ we impose that it is anti-symmetric respect to neighbour relative position *x*. Imposing anti-symmetrization makes the logit of the probability *p* change sign when we change the sign of the position *x*_*i*_ of the neighbour *i*. Then if the focal has a high probability *p* of moving to the right when a neighbour is at its right at, say, (*x*, *y*) = (5, 1), then the focal will react with a low probability of moving to the right, 1 − *p*, when the neighbour is at (*x*, *y*) = (− 5, 1) (or equivalently, with a high probability *p* of moving to the left).

SocialNet gives test accuracies similar to those of the interaction network and confirms that the more relevant variables of the neighbour are position and velocity (figure 7*b*). The advantage of SocialNet is that it consists of two functions of low dimensionality that we can plot and analyze.

### (d) How uninformed focal animals weight other animals, according to SocialNet

We are interested in how an uninformed focal weights the different neighbours. This is given by the aggregation function *W*. This function estimates the importance that the uninformed focal gives to a neighbour depending on focal and neighbour variables (focal speed, neighbour position and neighbour speed). There are 14 of these functions *W*, one per neighbour, but they are all identical functions. The output value of each of them can be different because the input to each of them can be different.

**Figure 8.**
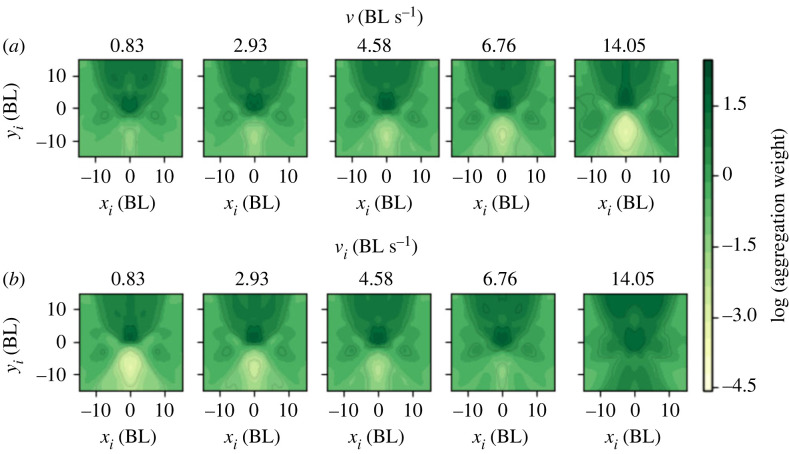
How uninformed focal weights neighbours. (*a*) Weight function *W* in the space defined by the neighbour position (*x*_*i*_, *y*_*i*_), for focal speeds of *v* = 0.83, 2.93, 4.58, 6.76 and 14.05 BL s^−1^ with neighbour at the median speed of *v*_*i*_ = 4.58 BL s^−1^. (*b*) As (*a*) but this time for different neighbour speeds of *v*_*i*_ = 0.83, 2.93, 4.58, 6.76 and 14.05 BL s^−1^ and with focal at the median speed of *v* = 4.58 BL s^−1^.

We can plot this weight function *W* in the space defined by the neighbour position (*x*_*i*_, *y*_*i*_). Figure 8*a* gives *W*(*x*_*i*_, *y*_*i*_) for the neighbour speed fixed at the median speed of *v*_*i*_ = 4.58 BL s^−1^. Each of the plots in this row is for a different speed of the focal: 0.83, 2.93, 4.58, 6.76 and 14.05 BL s^−1^. We can see in the plots that the faster the focal moves, the lesser importance it gives to fish behind.

Figure 8*b* gives *W*(*x*_*i*_, *y*_*i*_) for the focal speed fixed at the median speed of *v* = 4.58 BL s^−1^, and each of the plots in this row is for a different speed of the neighbour of values 0.83, 2.93, 4.58, 6.76 and 14.05 BL s^−1^. We can see in the plots that the focal gives more weight to all space for higher neighbour speeds. While neighbours are more important when in front, for the same neighbour position and focal velocity, the neighbour is more important the faster it is moving. For high neighbour speeds, importance is more independent of position.

**Figure 9.**
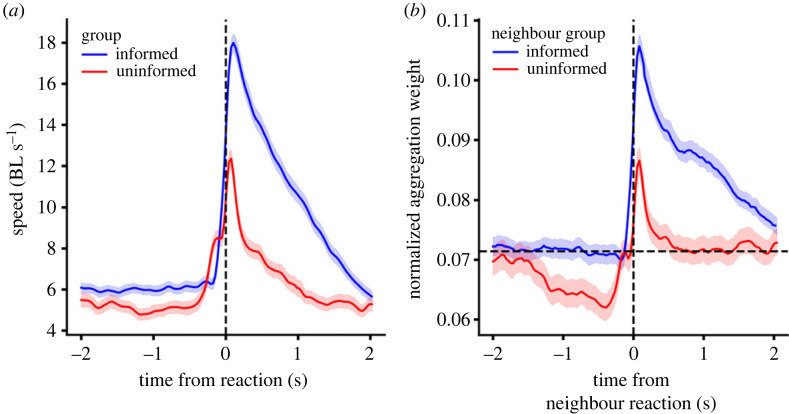
Mean speed of fish before and after the time of their reaction to light on. (*a*) Speed of informed and uninformed fish in mixed experiment, as a function of time from fish reaction. (*b*) Normalized weight, according to SocialNet, that an uninformed fish gives to informed and uninformed fish, depicted in time with 0 being the reaction to light turning on.

We can summarize our results as follows. When the light turns on, the informed animals react first within 1 s (figure 5*d*–*f*). Part of this reaction is that their speed increases from 6 BL s^−1^ to 18 BL s^−1^ (figure 9*a,* blue line). The uninformed fish at this point are giving a very high weight to informed fish because their speed is higher than that of uninformed fish according to the weight function in figure 8. Indeed, we can plot the normalized weight in time, and it increases sharply for informed animals when they react (from 0.07 before reaction to 0.1 after reaction and is maintained high for 2 s (figure 9*b*, blue line)). At this point, uninformed fish have not yet reacted and their normalized weight is below 0.07 (figure 9*b*, red line before reaction). After some of the informed fish have reacted, the uninformed fish turn towards them and move at higher speeds (figure 9*a*, red line). The uninformed fish continue weighting more those fish moving at higher speeds, which now can be both informed or uninformed (figure 9*b*).

## 4. Discussion

We have taken advantage of deep learning to increase the toolbox of analyses of animal collectives. We have built idmatcher.ai to follow animals across different videos, ReactNet to find when an animal is reacting to an external stimulus, and SocialNet as a model of interactions. We used the three methods together in an experiment designed to have a subset of animals following another subset of animals at a specific moment. We found support for the idea that the speed of neighbours is important in the transfer of information within a group. More specifically, we obtained how the decision of the focal depends on how neighbour speed interacts with other relevant variables, like focal speed and acceleration and neighbour relative location. However, when fixing the rest of variables, the higher the neighbour speed, the higher the impact of that neighbour. And at high enough neighbour speeds, the rest of the variables have a lesser effect in the focal decision.

We would like to put our results in context. Experimental designs similar to ours exist in the literature, e.g. golden shiners trained to expect food out of the shade and at a certain time of day could lead uninformed neighbours to the food source [40]. There are also results in the literature to support the importance of speed in animal interactions. Expressing interactions as forces, it has been found that they depend on non-trivial combinations of the neighbour’s position and velocity [41,42]. Experiments using robotic fish showed that the speed of the robot was important for the interactions with guppies [43]. Also, while most modelling work does not use speed as a relevant variable [44–51], the importance of speed has also been shown in the modelling work [52–54], including an impact in the collective emergent dynamics [55]. In the context of these results, SocialNet shows how decisions depend on two low-dimensional functions. These two functions describe how a few variables interact to explain decisions. Speed is one of these variables. Its effect on the attention module depends also on neighbour relative position and focal speed. Further, its effect in the final decision also depends on the focal acceleration perpendicular to the focal velocity vector and on the neighbour velocity vector (variables of the pair-interaction module, figure 7*b*). The effect of speed is thus quite nontrivial as it is coupled to other variables. In general, the higher the speed, the more important the neighbour in the decision, but its impact depends also on the other variables.

As SocialNet is a modelling approach that is more complex than the models traditionally used in the study of collective behaviour, we would like to clarify its properties with respect to overfitting and generalization. Simple analytic models contain few parameters and a strong *a priori* structure limiting which interaction functions can be learned. Deep learning models contain many parameters and are universal function approximators. The two classes of models sit in opposite extremes in the bias–variance trade-off. If the experimental dataset is small, the stronger inductive bias of analytic models can avoid overfitting and produce better models than deep learning approaches. In the limit of large experimental datasets, deep learning models behave better, while simple models underfit if their structure does not consider key effects contained in the data. A second consequence of the structure of deep learning models is that they preclude easy interpretation, particularly compared to simple models with few parameters and a theoretically motivated structure. Both classes of models sit in opposite extremes of the generally accepted accuracy–interpretability trade-off: but see [56]. SocialNet sits in a middle ground in both the bias–variance and accuracy–interpretability trade-offs by imposing a softer inductive bias of two types: biologically and physically motivated symmetries, and relational inductive biases that enable reasoning about structure and compositionality [57].

In practice, SocialNet can be used in different ways. One might want to give a detailed quantitative description of a given experiment or a set of experiments. If you have enough data, SocialNet is going to give you much more detail about your experiment than simpler models can. It can also have advantages with respect to traditional data analysis techniques thanks to the ability of networks to interpolate. However, one might be more interested in a comparison across species or conditions. SocialNet can also be used in this case similarly to how one would use data analysis methods. Simple models, however, if they can capture a common mechanism underlying different species or situations, might give you a more understandable comparison. For example, a simple model can have a parameter that explains differences across species when it takes different values. Simpler models have this extrapolation ability that complex models are very unlikely to have. The price that simple models pay for their extrapolation ability is that they likely miss some important factors. We propose to use SocialNet also to inform simpler models of which important factors might be missing. We can exemplify how this would work using one of our previously proposed simple models. We proposed to model animals in collectives as Bayesian agents that use the behaviours of others to make decisions [35,36]. From this idea, one can obtain simple analytical expressions by using additional assumptions. We used the assumption that it is the number of neighbours choosing one of several discrete options that gives information to the focal animal about that option being good. A single interpretable parameter in the model explained differences across species [35]. Yet, this model failed in dynamic situations without clear discrete options [58]. Given the results of SocialNet, it seems reasonable to think that the assumptions added to the Bayesian model were too strong for dynamic situations. Instead, speed could play a prominent role in the Bayesian model as a neighbour moving faster towards a location in space can indicate that a region of space has a higher probability of being a good option. One should therefore consider a rederivation of the Bayesian model with neighbour speed as a relevant variable, or maybe a simple coupling of neighbour speed and neighbour relative location consistent with that found in SocialNet.

ReactNet was an additional network that we used to understand our experiments. ReactNet determines when each fish responds to light. This was used to validate our experimental setup, showing that, as expected, the fish that first respond to the light are the trained ones. ReactNet was also necessary to show that the naïve fish react to the trained fish at different times.

Our results can be extended in several directions. Without any further development, our proposed tools can be used in scenarios different from ours. idmatcher.ai can be used in the lab to follow a subset of animals in a collective that is different for some reason. This can include manipulations of their physiology, different experiences, or any other differences (circadian rhythms, sex, etc). ReactNet can be used for any external stimulus one might want to use, and not only for light. SocialNet can be used for variables very different from the ones we used. For example, one might want to consider the possible importance of neighbour postures. Extensions of SocialNet could consider more than one output to produce not only a point-prediction but also trajectories, e.g. in a reinforcement learning setup. Larger experimental datasets will allow deep learning techniques to model the behaviour of animals with increasing detail. We will likely see techniques that ultimately can give you a mathematical avatar of each of the animals in a group and follow them in their development and interactions with others and with the world. While in principle these methods might be thought to be very data hungry, this problem might be reduced using transfer learning [21,22,59] as a starting point in models as well as using a combination of unsupervised and supervised methods [24]. Also, it will soon be possible to combine this behavioural modelling with different types of datasets, including measurements of brain activity, internal states and details of the environment.

## Data Availability

Data and code are available at https://gitlab.com/polavieja_lab/idmatcherai.

## Endnote

1 https://docs.scipy.org/doc/scipy/reference/generated/scipy.optimize.linear_sum_assignment.html.
